# Frailty-Adjusted Inpatient Glycaemic Targets for Preventing Hypoglycaemia: A Quality Improvement Project

**DOI:** 10.7759/cureus.100238

**Published:** 2025-12-28

**Authors:** Hein Htet Zaw, Warda Mansur, Jesslin Austin, Laju Gurung, Kate Dean

**Affiliations:** 1 Internal Medicine, Royal Berkshire Hospital, Reading, GBR; 2 Geriatrics, Royal Berkshire Hospital, Reading, GBR

**Keywords:** frailty, glycaemic targets, hypoglycaemia, inpatient care, older adult diabetes

## Abstract

Background: Older adults with frailty are particularly vulnerable to harm from tight glycaemic control, with hypoglycaemia contributing to falls, cognitive decline, and increased mortality. National (JBDS: Inpatient Care of the Frail Older Adult with Diabetes) and local (Buckinghamshire, Oxfordshire & Berkshire-BOB) guidelines recommend individualising glucose targets according to frailty level, but adherence is inconsistent. This quality improvement project evaluated glycaemic management in inpatients with frailty, aiming to reduce hypoglycaemia and improve target setting in line with frailty status.

Methodology: A two-cycle quality improvement project was conducted across four elderly-care wards at the Royal Berkshire Hospital. The inclusion criteria included patients aged ≥65 years with a diagnosis of either Type 1 or Type 2 diabetes who experienced one or more hypoglycaemic episodes (<4 mmol/L) during their admission. Ward-level hypoglycaemia incidence was calculated as the proportion of all elderly-care inpatients aged ≥65 with diabetes who experienced at least one hypoglycaemic episode during each cycle. Further analyses were performed only within the cohort of patients who experienced hypoglycaemia. Cycle 1 (January to March 2025) included 31 patients meeting these criteria out of 209 elderly-care inpatients aged ≥65 with diabetes (15%). Cycle 2 (May to July 2025) included 12 out of 202 patients (6%) following staff education and reinforcement of local hypoglycaemia management guidelines. Frailty was assessed using the Clinical Frailty Scale (CFS), and glucose targets were set according to frailty category. Data from Diabetes Specialist Nurse referrals and incident reports were analysed in both cycles to assess hypoglycaemia rates, documentation of glucose targets, and medication adjustments.

Results: Between cycles, the incidence of hypoglycaemia in elderly inpatients with diabetes decreased by 60% (from 15% to 6%). Among those who experienced hypoglycaemia, the proportion with frequent episodes (≥3 episodes) fell by 10% (from 77% to 67%). The incidence of significant hypoglycaemia (≤3 mmol/L) remained unchanged at 58%. Among patients with a CFS of 7-9, correct glucose target documentation improved substantially, rising from 25% to 80%. In contrast, documentation fell from 56% to 20% in those with a CFS of four to six, showing variability in practice. Proactive medication optimisation, such as insulin de-escalation and adjustment of oral hypoglycaemic agents, was carried out more consistently in Cycle 2.

Conclusions: Frailty-adjusted glycaemic targets and proactive medication review can markedly reduce hypoglycaemia in older adults with frailty during their admission. Sustaining improvements will require ongoing staff education, standardised pathways, and clear documentation to ensure safer diabetes management in this vulnerable population.

## Introduction

Older adults with diabetes represent a particularly vulnerable population, and this vulnerability is markedly amplified when frailty complicates disease management. Frailty, defined as diminished physiological reserve and increased susceptibility to stressors, is highly prevalent among individuals with long-standing diabetes and multiple comorbidities [1].

Two phenotypic patterns of frailty are commonly described in older adults with diabetes: a sarcopenic phenotype, characterised by loss of muscle mass and strength, and an obesity-associated phenotype, often referred to as sarcopenic obesity, in which excess adiposity coexists with reduced muscle quality and impaired metabolic reserve. Both phenotypes contribute to functional decline and greater vulnerability to hypoglycaemia and other adverse outcomes [2].

Diabetes and frailty also have a bidirectional relationship. Long-standing diabetes increases the risk of frailty through mechanisms such as chronic inflammation, neuropathy, vascular disease, and accelerated sarcopenia, while frailty increases susceptibility to hypoglycaemia due to impaired counter-regulatory responses, polypharmacy, inconsistent nutritional intake, and reduced physiological reserve [3].

Recognising frailty early, ideally at the point of hospital admission, is essential. Evidence shows that inpatients assessed as moderately or severely frail are at significantly higher risk of adverse outcomes, including recurrent hypoglycaemia, falls, cognitive decline, and hospital readmissions [4,5]. Moreover, frequent and severe hypoglycaemic episodes among frail patients have been associated with increased mortality independently of age alone [6].

Frailty screening can be a practical and feasible component of inpatient diabetes management. The Rockwood Clinical Frailty Score (CFS) or similar validated frailty tools can be applied promptly on admission, enabling stratification of patients into non-frail, moderately frail, and severely frail categories. This stratification informs personalised glycaemic targets and care plans [7]. In the inpatient setting, such an approach supports early identification of patients at greatest risk of hypoglycaemic events and allows tailored interventions to mitigate this risk.

Hypoglycaemia presents a major challenge in older adults with diabetes and frailty. The combination of polypharmacy, impaired counter-regulatory mechanisms, and inconsistent nutritional intake further heightens this risk [8]. Importantly, overly stringent glycaemic targets, often derived from standard management approaches, can precipitate frequent and severe hypoglycaemic episodes, particularly in the moderately and severely frail inpatient group, thereby further increasing mortality risk [9]. Consequently, leading guidance from organisations such as the Joint British Diabetes Societies for Inpatient Care (JBDS-IP) and the Buckinghamshire, Oxfordshire and Berkshire Integrated Care Board (BOB ICB) now advocate for individualised glycaemic targets based on frailty, comorbidities, and life expectancy [10]. However, adherence to these recommendations remains inconsistent in clinical practice due to variability in staff awareness and confidence in applying frailty-specific targets [11].

The primary objective of this Quality Improvement Project (QIP) was to determine the prevalence of hypoglycaemia among frail older inpatients with diabetes. The secondary objective was to evaluate whether targeted staff education and the implementation of a frailty-based glycaemic target flowchart could reduce the incidence of hypoglycaemic episodes. The project sought to enhance patient safety by aligning inpatient practice with national guidance and promoting individualised, frailty-focused diabetes care.

This project was previously presented as a poster at the G4J Conference 2025.

## Materials and methods

Project design and setting

**Figure 1:**
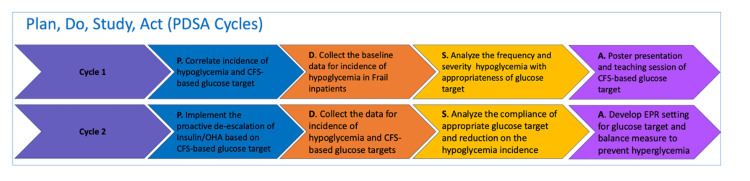
Plan–Do–Study–Act (PDSA) cycle applied in the quality improvement project This figure illustrates the Plan–Do–Study–Act (PDSA) methodology applied across two cycles of QIP to evaluate and improve inpatient glycaemic management among older adults with frailty. The model depicts the iterative process used to implement staff education, reinforce individualized glycaemic target setting, and assess outcomes.

This retrospective, two-cycle QIP was conducted in four elderly care wards of the Royal Berkshire NHS Foundation Trust, an 800-bedded district general hospital in the UK, between January and July 2025. The project followed the Plan-Do-Study-Act (PDSA) methodology to evaluate and improve inpatient diabetes management among frail older adults. The primary aim was to reduce the frequency and severity of hypoglycaemia by enhancing staff awareness and promoting individualised glycaemic target setting based on frailty status. A visual summary of the PDSA process used throughout the project is shown in Figure 1.

Inclusion and exclusion criteria

Diabetes diagnosis was confirmed through documentation in the electronic medical record supported by laboratory investigations, including historical or admission HbA1c, fasting or random plasma glucose measurements, or existing diagnostic coding consistent with NICE diagnostic criteria. Participants were selected according to the predefined inclusion and exclusion criteria presented in Table 1.

**Table 1:** Inclusion and exclusion criteria for study participants Hypoglycaemia is defined as CBG < 4.0 mmol/L. CBG: capillary blood glucose.

Criterion	Inclusion Criteria	Exclusion Criteria
Age	≥65 years	<65 years
Admission type	Elderly care wards	Other wards
Type of diabetes	Confirmed diagnosis of diabetes mellitus (Type 1 or Type 2)	Transient steroid-induced hyperglycaemia (no underlying diabetes)
Hypoglycaemia	At least one documented hypoglycaemic episode (CBG <4.0 mmol/L) during inpatient stay	No documented hypoglycaemic episodes during admission

These criteria were applied consistently in Cycle 1 (retrospective) and Cycle 2 (prospective)

Frailty assessment

Frailty was assessed using the Rockwood Clinical Frailty Scale (CFS), a nine-point ordinal scale ranging from 1 (Very Fit) to 9 (Terminally Ill). Patients with a CFS of 4-6 were classified as mildly to moderately frail, while those with a CFS of 7-9 were categorised as severely frail. This tool provides a structured assessment of physiological reserve and vulnerability, allowing stratification into clinically meaningful frailty categories.

Cycle 1: baseline data collection and analysis

Baseline data were collected retrospectively from January to March 2025. Eligible inpatients were identified through DSN referrals and Datix incident reports. Data were extracted from electronic medical records and DSN databases and included demographic characteristics (age and sex), type of diabetes, relevant comorbidities (including chronic kidney disease and sepsis), insulin regimen, use of oral hypoglycaemic agents (OHAs), and CFS score.

Continuous glucose monitoring (CGM) was not routinely used for inpatient care during the study period; therefore, all hypoglycaemic events were identified using point-of-care capillary blood glucose measurements recorded in nursing documentation and DSN referrals.

The total number of eligible patients was determined using information provided by the hospital informatics and administrative team, based on admission ward, admission date within the data collection period, age ≥65 years, and a confirmed diagnosis of Type 1 or Type 2 diabetes mellitus.

Patients were stratified according to frailty status using the Rockwood CFS. The incidence of frequent hypoglycaemia (≥3 episodes of hypoglycaemia per inpatient stay) and significant hypoglycaemia (capillary blood glucose ≤3.0 mmol/L) was calculated.

The analysis explored associations between hypoglycaemia, frailty level, and appropriateness of glucose targets based on frailty status. Documentation of appropriate glycaemic targets was reviewed to identify cases with overly tight glucose control or missing target documentation. Proactive medication optimisation, such as simplification or de-escalation of insulin therapy and deprescribing of OHAs based on patient comorbidities and frailty, was assessed to determine whether appropriate adjustments were made.

Intervention

Following the baseline analysis, findings were disseminated across the Trust through multiple multidisciplinary forums to promote shared learning and system-wide improvement. Results were presented at the Insulin Safety Committee Meeting, the Elderly Care Clinical Governance Meeting, and during departmental teaching sessions for resident doctors.

The educational intervention emphasised the routine use of the CFS to assess frailty on admission, the importance of setting appropriate and individualised glycaemic targets for older adults with diabetes and frailty, and the need for alignment with recommendations from the Joint British Diabetes Societies (JBDS) and the Buckinghamshire, Oxfordshire and Berkshire Integrated Care Board (BOB ICB).

**Figure 2:**
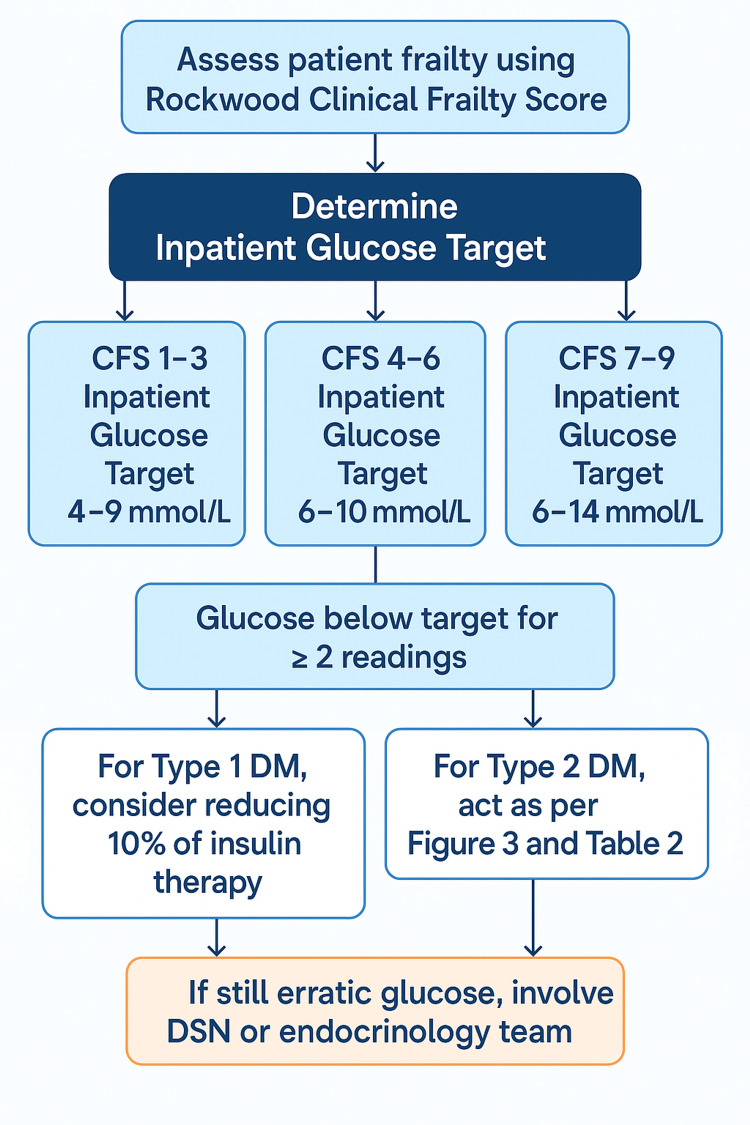
Flowchart for setting inpatient glucose targets based on frailty This flowchart outlines a stepwise approach for determining appropriate inpatient glucose targets according to the Rockwood Clinical Frailty Scale (CFS). Patients are stratified into three frailty categories (CFS 1–3, CFS 4–6, and CFS 7–9), each assigned a corresponding glucose target range. Subsequent actions are recommended for patients with glucose levels falling below target on two or more readings, including insulin dose reduction for type 1 diabetes mellitus and reference to guideline algorithms for type 2 diabetes mellitus. Persistent glycaemic instability prompts involvement of the diabetes specialist nurse (DSN) or endocrinology team.

**Figure 3:**
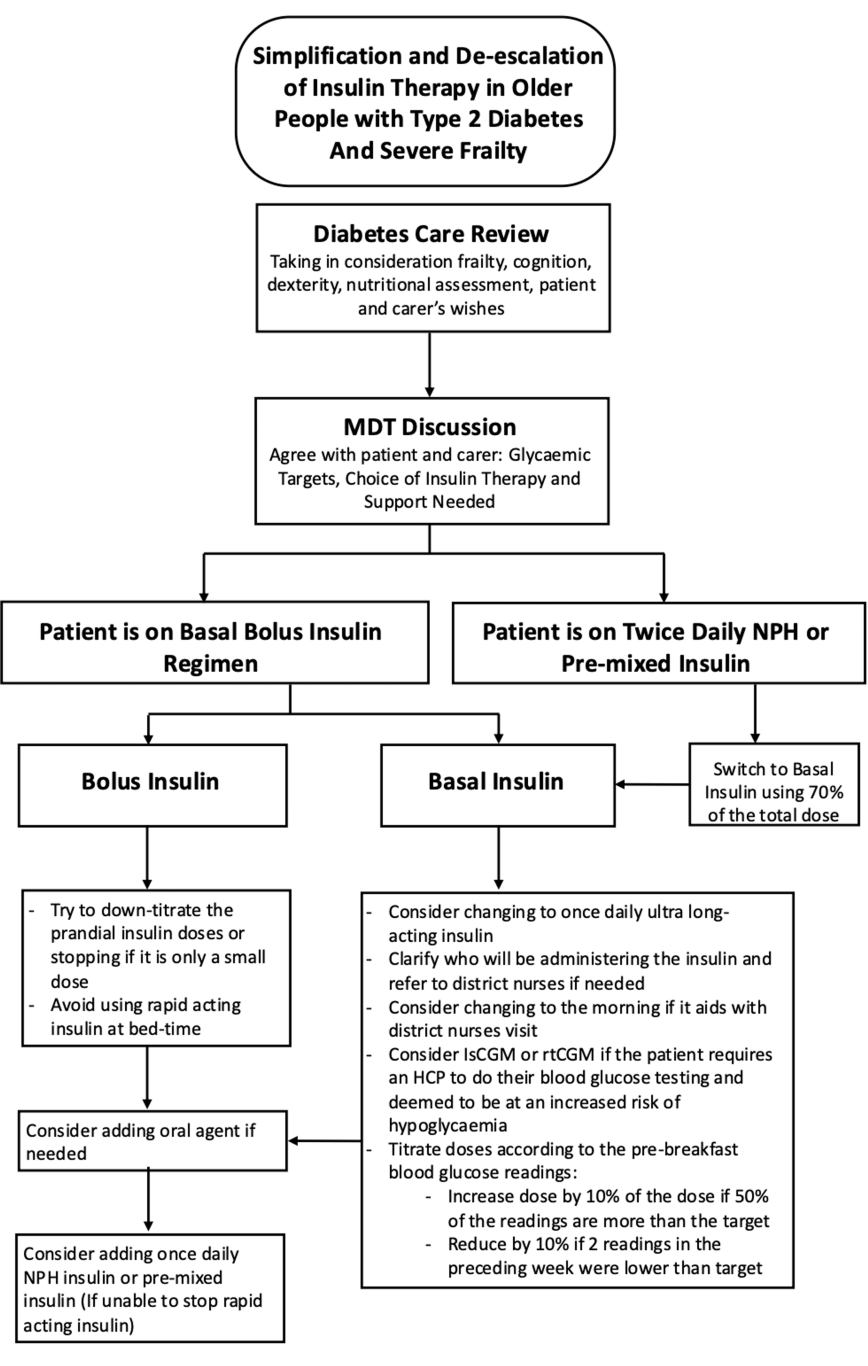
Simplification and de-escalation of therapy framework in people with Type 2 diabetes with insulin deficiency and severe frailty. This figure summarises the principles guiding medication adjustments in frail adults with diabetes, including recommendations for insulin simplification. MDT: multidisciplinary team, NPH: neutral protamine Hagedorn, IsCGM:  intermittently scanned continuous glucose monitoring, rtCGM: real-time continuous glucose monitoring, HCP: healthcare professionals.

Practical examples were used to demonstrate frailty recognition, appropriate adjustment of glucose targets, and therapy modification. Clinicians were encouraged to simplify insulin regimens, de-escalate therapy when appropriate, and avoid unnecessary intensification. Ward posters and visual reminders summarising frailty-specific glucose management principles were displayed in clinical areas. Examples of these materials are shown in Figures 2, 3 and Table 2.

**Table 2:** Pros and cons of anti-hyperglycaemic therapies for the treatment of type 2 diabetes in older adults This figure presents a comparative overview of commonly used antihyperglycemic medications, highlighting relative risks of hypoglycaemia, treatment burden, and suitability in the context of frailty for proactive treatment de-escalation. CV: cardiovascular, CA: cancer, DDP4: dipeptidyl peptidase 4, SGLT2: sodium-glucose cotransporter 2, GLP-1 RA: glucagon-like peptide-1 receptor agonist, TZD: thiazolidinedione.

Anti-hyperglycaemic Therapy	Pros	Cons
Metformin	• Well-established, generally well-tolerated standard therapy • Low hypoglycaemia risk • Can be combined with all other diabetes therapies	• Reduced appetite, gastrointestinal disturbance, possible B12 deficiency • Contraindicated in severe renal failure • Caution in hepatic/cardiac impairment
Sulphonylureas	• Can be combined with other therapies • Increased potency in older adults	• Hypoglycaemia risk, especially after weight loss
DDP-4 inhibitors	• Well-tolerated • Low risk of hypoglycaemia • No effect on weight	• Low to moderate glucose-lowering efficacy • Possible increased risk of hospitalization for HF with Saxagliptin (± alogliptin)
SGLT-2 inhibitors	• Benefits for diabetes with heart failure and renal impairment • Low hypoglycaemia risk	• Genital candidiasis, urinary incontinence • Lack of efficacy in severe renal impairment • Risk of diabetes ketoacidosis • Fluid volume depletion
GLP-1 RAs	• Reno-protective effects • Low hypoglycaemia risk with good efficacy	• Weight loss may worsen sarcopenia • Possible worsening of diabetic eye disease with Semaglutide
TZDs	• Generally, well tolerated • Low hypoglycaemia risk	• Fluid retention may worsen heart failure • Risk of osteoporotic fractures and bladder cancer

Cycle 2: post-intervention data collection

Following a one-month consolidation period after the intervention, data for Cycle 2 were collected prospectively between May and July 2025 using the same inclusion criteria and data sources as in Cycle 1, which was collected retrospectively. This phase aimed to evaluate changes in clinical practice and patient outcomes resulting from the educational and procedural interventions introduced during the first cycle.

Cycle 2 analysis focused on assessing whether the implementation of staff education and frailty-based glucose management led to a reduction in both the frequency and severity of hypoglycaemia. In addition, the documentation of individualised glycaemic targets based on the CFS was reviewed to determine improvements in adherence to national guidance. Evidence of appropriate medication adjustments, such as insulin de-escalation or deprescription of OHAs among frail patients, was also examined to assess clinical responsiveness to hypoglycaemia risk.

Outcome measures

The primary outcome of this project was the change in hypoglycaemia rates, both in terms of frequency and severity, between the two audit cycles. Secondary outcomes included the proportion of patients with clearly documented individualised glycaemic targets according to their frailty level and the rate of medication de-escalation among moderately and severely frail patients. The project also evaluated overall staff adherence to Joint British Diabetes Societies (JBDS) and Buckinghamshire, Oxfordshire and Berkshire Integrated Care Board (BOB ICB) recommendations for inpatient diabetes management documentation.

Data analysis

Descriptive statistics were used to compare data between cycles. Categorical variables, such as the presence of individualised target documentation and medication de-escalation, were presented as percentages, while continuous variables, such as the number of hypoglycaemic episodes, were expressed as means or medians where appropriate. Comparative evaluation focused on observed trends in improvement rather than inferential statistical testing, given the small sample size typical of QIP designs.

Ethical considerations

This project was registered as a local QIP with the Royal Berkshire NHS Foundation Trust’s audit and governance department. As no patient-identifiable data were disclosed and the project involved evaluation of existing care processes, formal ethical approval was not required in accordance with NHS Health Research Authority guidance.

## Results

First PDSA cycle (January-March 2025)

During the first PDSA cycle, 31 patients were included in the baseline assessment, identified from a total of 209 inpatients aged >65 years with either Type 1 or Type 2 diabetes. The overall incidence of hypoglycaemia during this period was 15%.

Frailty was highly prevalent in this cohort: 26 of the 31 patients (84%) were classified as having mild to moderate or severe frailty, reinforcing frailty as one of the most significant risk factors for inpatient hypoglycaemia.

Assessment of glycaemic target documentation revealed notable inconsistencies. Among patients with CFS 4-6, 56% (10 out of 18) had appropriate individualised glucose targets documented. However, documentation was markedly poorer among severely frail patients (CFS 7-9), with only 20% (2 out of 8) having individualised targets recorded. These findings highlighted a substantial gap in the application of frailty-aligned glycaemic management and underscored the need for improved awareness, documentation, and adherence to individualised inpatient glucose targets, forming the foundation for subsequent educational and procedural interventions.

Second PDSA cycle (May-July 2025)

The second PDSA cycle included 12 patients out of 202 eligible inpatients aged >65 years with diabetes, indicating an incidence rate of 6%. The overall incidence of hypoglycaemia fell markedly from 15% in Cycle 1 to 6% in Cycle 2, representing a 60% reduction. This improvement was most evident among frail patients, where the introduction of individualised glycaemic targets and closer monitoring appeared to reduce the frequency and severity of hypoglycaemic episodes.

Similarly, 10 of the 12 patients (83%) in Cycle 2 had a CFS score of ≥4, reaffirming that frailty remains the most common risk factor for inpatient hypoglycaemia.

**Figure 4:**
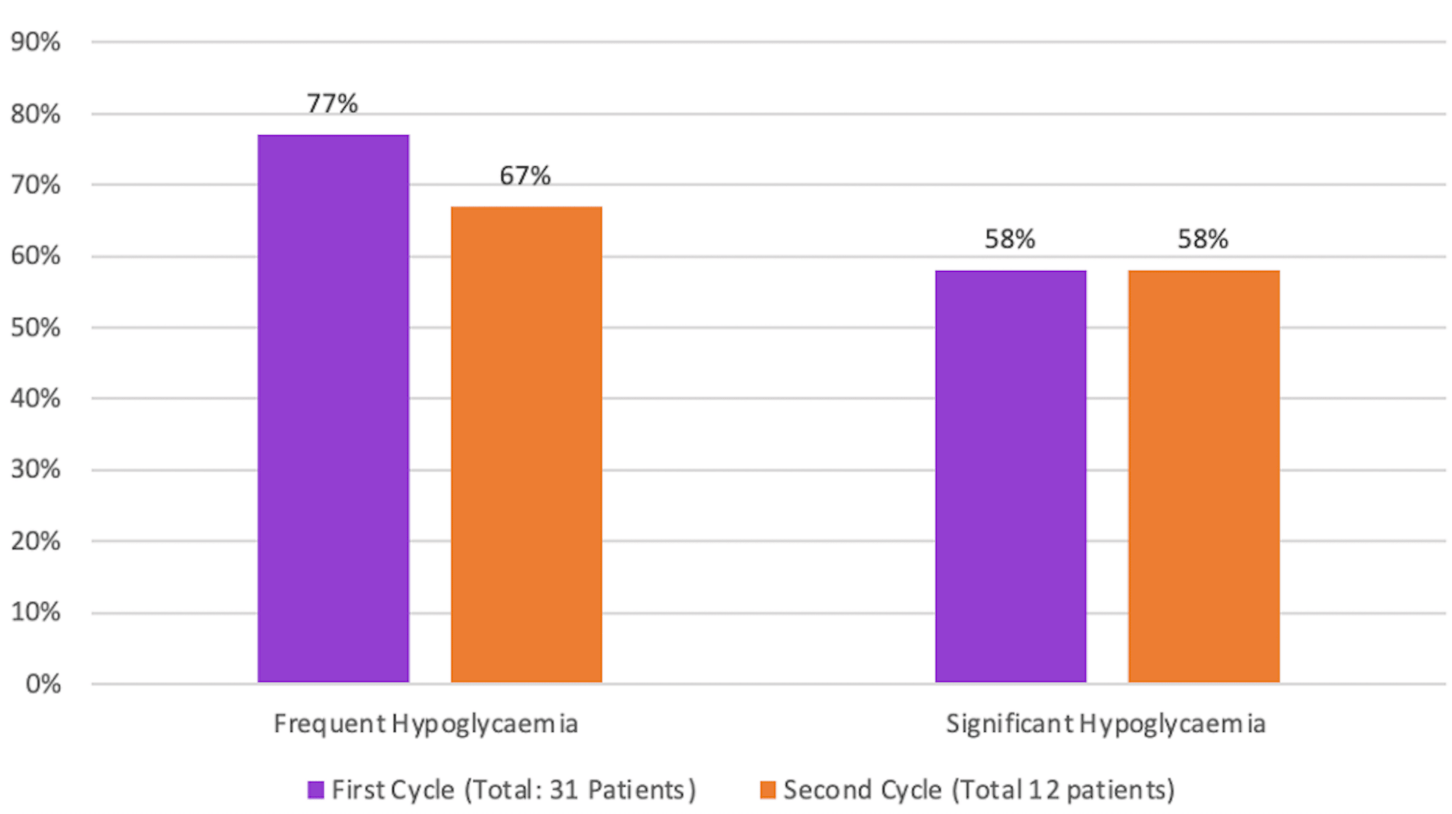
Incidence of frequent and significant hypoglycaemia between two audit cycles This bar chart compares the proportion of patients experiencing frequent hypoglycaemia (≥3 episodes per admission) and significant hypoglycaemia (capillary glucose ≤3.0 mmol/L) in Cycle 1 and Cycle 2. The figure demonstrates a reduction in frequent episodes following the intervention, with rates of significant hypoglycaemia remaining stable.

Frequent hypoglycaemia (≥3 episodes per admission) decreased from 77% (24 of 31 patients) in Cycle 1 to 67% (8 of 12 patients) in Cycle 2. In contrast, the rate of significant hypoglycaemia (capillary blood glucose ≤3.0 mmol/L) remained stable at 58% across both cycles (18 of 31 in Cycle 1 vs. 7 of 12 in Cycle 2), as shown in Figure 4. This stability may reflect the persistence of complex clinical contributors, such as multimorbidity, fluctuating nutritional intake, and increased insulin sensitivity, which remain challenging to fully mitigate despite enhanced monitoring.

**Figure 5:**
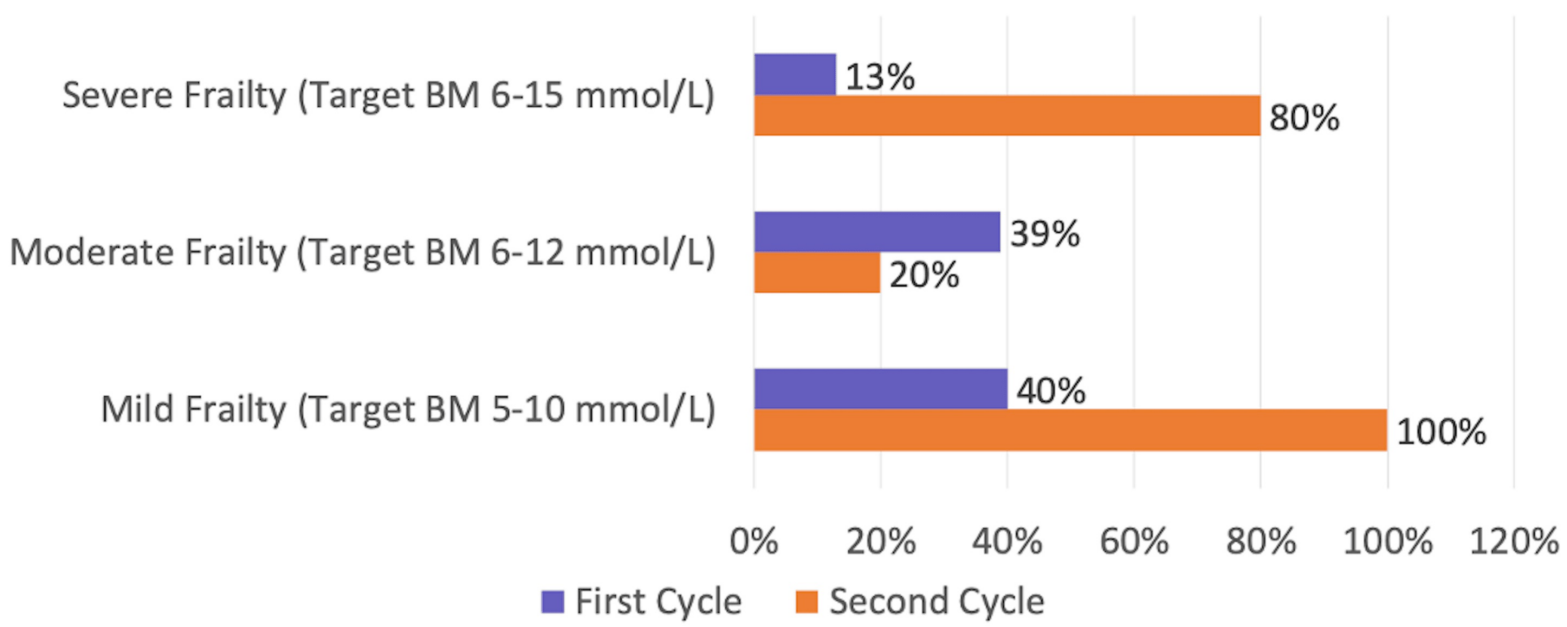
Appropriate inpatient glucose targets based on frailty category This figure displays the percentage of patients in each frailty group (CFS 4–6 and CFS 7–9) who had correctly documented individualized inpatient glucose targets in Cycle 1 and Cycle 2. Marked improvement is observed in the severely frail cohort following the intervention. CFS: Clinical Frailty Scale, BM: blood glucose monitoring.

Documentation of frailty and individualised glucose targets improved substantially following the intervention. Among patients with severe frailty (CFS 7-9), appropriate documentation increased from 25% at baseline to 80% post-intervention. However, the proportion of mildly or moderately frail patients (CFS 4-6) with correct target setting declined from 56% to 20%. Figure 5 illustrates the distribution of appropriate glucose target documentation by frailty level.

Medication management practices also showed notable improvement. During the first cycle, insulin de-escalation and the deprescription of OHAs were inconsistent. In the second cycle, these practices were applied more systematically, with clinicians demonstrating greater awareness of the need to reduce overtreatment and align pharmacological management with individualised glycaemic goals. The findings suggest an overall shift towards safer, evidence-based prescribing practices in the management of frail inpatients with diabetes.

## Discussion

This two-cycle QIP demonstrated that adopting frailty-based glycaemic targets and enhancing staff education substantially reduced hypoglycaemia among frail inpatients with diabetes. A 60% reduction in overall hypoglycaemia and a 10% decrease in recurrent episodes highlight the impact of structured education and systematic practice change. These findings align with existing evidence showing that most inpatient hypoglycaemic events are preventable through individualised management, medication review, and avoidance of overtreatment [12,13]. International consensus also emphasises the importance of aligning inpatient diabetes care with frailty status, particularly to reduce the risks associated with overly stringent glycaemic control in older adults [14].

Documentation of individualised glycaemic targets improved markedly among severely frail patients (CFS 7-9), reflecting enhanced engagement with national and local diabetes guidelines. This is consistent with international recommendations, which highlight the need to tailor glycaemic thresholds to functional status and frailty severity to reduce harm and improve outcomes [15]. The decline in correct target setting among mildly or moderately frail patients (CFS 4-6) suggests that further reinforcement of frailty-specific care across all categories is needed. Embedding frailty scoring and individualised target prompts within electronic health records may help standardise practice and sustain improvement.

Medication optimisation also strengthened following the intervention, with more consistent deprescribing of sulfonylureas and insulin de-escalation, approaches aligned with international recommendations prioritising safety over tight glycaemic control [16]. Ward-based visual reminders appeared to improve uptake of these principles. Ensuring recent HbA1c values are within target before reducing treatment remains critical to avoid rebound hyperglycaemia.

Current guidelines emphasise that glycaemic targets for frail older adults should prioritise safety and avoidance of hypoglycaemia rather than tight control. Evidence from JBDS-IP and international consensus statements suggests that a target capillary blood glucose range of 6-10 mmol/L is generally appropriate for moderately frail patients (CFS 4-6), while a broader range such as 6-14 mmol/L may be safer for severely frail or anorexic, malnourished patients (CFS 7-9), in whom glucose variability and impaired counter-regulatory mechanisms substantially heighten the risk of hypoglycaemia. These more permissive targets limit unnecessary insulin titration and help stabilise blood glucose profiles.

Choice of hypoglycaemic therapy is also critical in frail, anorexic, or malnourished older adults. Agents with minimal hypoglycaemia risk, such as DPP-4 inhibitors, low-dose basal insulin, or discontinuation of sulfonylureas, are preferred. Sulfonylureas and prandial insulin should be avoided or deprescribed in patients with poor oral intake, sarcopenia, or cognitive decline, as these substantially increase the likelihood of unpredictable hypoglycaemia. In undernourished frail patients, simplified regimens, for example, basal-only insulin or DPP-4 monotherapy, aligned with relaxed glycaemic targets, can prevent hypoglycaemia while maintaining safe glycaemic control.

Analysis of Cycle 2 patients with recurrent hypoglycaemia showed that, in many cases, episodes were driven by acute clinical stressors such as acute kidney injury (AKI) on chronic kidney disease (CKD) or sepsis, despite dose reductions. This reflects well-recognised patterns in frail older adults, in whom acute illness significantly increases insulin sensitivity and predisposes to hypoglycaemia [17]. Notably, patients with appropriately individualised targets experienced milder hypoglycaemia, supporting the role of frailty-adjusted thresholds in reducing both incidence and severity. Broader frailty frameworks also emphasise the need for early identification of physiological vulnerability to prevent adverse glycaemic outcomes [18].

Overall, the downward trend in hypoglycaemia underscores the effectiveness of this pragmatic, education-based approach. These improvements are aligned with global calls to action advocating for frailty-informed diabetes management to enhance safety, individualisation of care, and clinical outcomes [15,18]. Future work should focus on prospective, multi-ward implementation and electronic audit tools to support sustained quality improvement.

Limitations

This project had several limitations. The sample size was relatively small, and the study duration was short, which may limit the generalisability of the findings. Data collection was conducted retrospectively, introducing the possibility of reporting bias due to reliance on the accuracy and completeness of clinical documentation. In addition, potential confounding factors, such as variations in comorbidities, concurrent infections, and medication adjustments, were not fully controlled for, which may have influenced the observed outcomes.

Future work should focus on prospective, multi-ward implementation of frailty-based glycaemic management pathways, supported by electronic audit dashboards to enable real-time monitoring and continuous quality improvement across inpatient settings. Furthermore, the findings may have limited generalisability to other hospital settings due to variation in staffing, case mix, and local diabetes pathways.

## Conclusions

The implementation of frailty-based glycaemic targets and staff education within elderly care wards resulted in a measurable reduction in hypoglycaemia and improved documentation of individualised management plans. The CFS proved valuable in tailoring glucose targets, optimising medication regimens, and enhancing clinician awareness of safe diabetes care in vulnerable older adults.

To maintain these improvements, frailty-guided diabetes management should be embedded into institutional policy and electronic record systems. Broader, multi-centre adoption of this approach, supported by regular education and audit cycles, could further enhance patient safety and reduce hypoglycaemia-related harm across inpatient settings. This project underscores the value of integrating frailty assessment into routine diabetes care to achieve individualised, compassionate, and evidence-based management.
